# A wild boar cathelicidin peptide derivative inhibits severe acute respiratory syndrome coronavirus-2 and its drifted variants

**DOI:** 10.1038/s41598-023-41850-7

**Published:** 2023-09-05

**Authors:** Troy von Beck, Karla Navarrete, Nicholas A. Arce, Mu Gao, Gordon A. Dale, Meredith E. Davis-Gardner, Katharine Floyd, Luis Mena Hernandez, Nikita Mullick, Abigail Vanderheiden, Ioanna Skountzou, Suresh V. Kuchipudi, Rathi Saravanan, Renhao Li, Jeffrey Skolnick, Mehul S. Suthar, Joshy Jacob

**Affiliations:** 1 Emory Vaccine Center, Emory National Primate Research Center, Emory University, 954 Gatewood Road, Atlanta, GA 30329 USA; 2 Department of Pediatrics, Aflac Cancer and Blood Disorders Center, Children’s Healthcare of Atlanta, Emory University School of Medicine, Atlanta, GA 30322 USA; 3 Division of Infectious Diseases, Department of Pediatrics, Emory University School of Medicine, Atlanta, GA 30322 USA; 4 Animal Diagnostic Laboratory, Department of Veterinary and Biomedical Sciences, The Center for Infectious Disease Dynamics, Pennsylvania State University, University Park, PA 16802 USA; 5 Centre of Regulatory Excellence (CoRE), Duke-NUS Medical School, Level 6, 8 College Road, Singapore, 169857 Singapore; 6 Center for the Study of Systems Biology, School of Biological Sciences, Georgia Institute of Technology, 950 Atlantic Drive, NW, Atlanta, GA 30332 USA

**Keywords:** Viral infection, Antimicrobial responses

## Abstract

The severe acute respiratory syndrome coronavirus 2 (SARS-CoV-2) poses a clear threat to humanity. It has infected over 200 million and killed 4 million people worldwide, and infections continue with no end in sight. To control the pandemic, multiple effective vaccines have been developed, and global vaccinations are in progress. However, the virus continues to mutate. Even when full vaccine coverage is achieved, vaccine-resistant mutants will likely emerge, thus requiring new annual vaccines against drifted variants analogous to influenza. A complimentary solution to this problem could be developing antiviral drugs that inhibit SARS CoV-2 and its drifted variants. Host defense peptides represent a potential source for such an antiviral as they possess broad antimicrobial activity and significant diversity across species. We screened the cathelicidin family of peptides from 16 different species for antiviral activity and identified a wild boar peptide derivative that inhibits SARS CoV-2. This peptide, which we named Yongshi and means warrior in Mandarin, acts as a viral entry inhibitor. Following the binding of SARS-CoV-2 to its receptor, the spike protein is cleaved, and heptad repeats 1 and 2 multimerize to form the fusion complex that enables the virion to enter the cell. A deep learning-based protein sequence comparison algorithm and molecular modeling suggest that Yongshi acts as a mimetic to the heptad repeats of the virus, thereby disrupting the fusion process. Experimental data confirm the binding of Yongshi to the heptad repeat 1 with a fourfold higher affinity than heptad repeat 2 of SARS-CoV-2. Yongshi also binds to the heptad repeat 1 of SARS-CoV-1 and MERS-CoV. Interestingly, it inhibits all drifted variants of SARS CoV-2 that we tested, including the alpha, beta, gamma, delta, kappa and omicron variants.

## Introduction

Severe acute respiratory syndrome coronavirus 2 (SARS-CoV-2) is the newest member of the human Beta-coronavirus group and the causative agent of “coronavirus infectious disease 2019” (COVID-19), a disease characterized often by life-threatening viral pneumonia. SARS-CoV-2 shares over 96% genome sequence identity with a known bat coronavirus, suggesting a recent zoonotic transmission event^1^. Since first emerging in China, SARS-CoV-2 has spread globally and diverged into multiple variants with mutations permitting enhanced transmissibility and even escape from neutralizing antibodies generated by previous infection or vaccination with the original A.1 strain and A.1 spike-based vaccines^2^. The continued spread of SARS-CoV-2 and the potential emergence of highly transmissible variants of concern and eventually a fully vaccine-resistant strain mandates the need for new therapeutic antivirals in addition to ongoing vaccine improvements.

Plants and insects lack an adaptative immune system, but they can effectively fight microbial invasion by producing host defense peptides. In 1981, Hans Boman and colleagues demonstrated that the silkworms survive bacterial infection because of a peptide in the worms’ hemolymph. This was the first isolated host defense peptide, which they called cecropin^3^. Following this discovery, many other similar peptides that were discovered previously were identified as host defense peptides. Some of the most notable examples include human lysozyme discovered by Alexander Fleming in the 1920s, the peptide melittin isolated from bee venom, magainins isolated from the frog *Xenopus laevis,* as well as a family of peptides released from the granules of neutrophils that have broad-spectrum antimicrobial and antiviral capabilities called defensins^4–8^. In 1988 and 1989, the field of host defense peptides broadened with the elegant discovery of the antibacterial bactenecins Dodecapeptide, Bac-5, and Bac-7 in the cytoplasmic granules of bovine neutrophils^9,10^. Later efforts to capture the full-length cDNA of Bac-5 would serendipitously discover that Dodecapeptide, Bac-5, and Bac-7 all contained the same N-terminal pro-region with high sequence similarity to porcine cathelin^11,12^.

Dodecapeptide, Bac-5, and Bac-7, along with many other examples from mammals, birds, and some fish belong to the family of host defense peptides called cathelicidins, which possess broad antimicrobial activity^13–15^. This class of peptide is characterized by its pre-pro peptide structure formed by an N-terminal signal peptide and C-terminal antimicrobial domain flanking a highly conserved cathelin-like domain. This organization permits the synthesis and storage of cathelicidins in an inert form until proteolytic processing during degranulation or phagocytosis releases the C-terminal antimicrobial domain (hereafter referred to as “cathelicidin peptide”). Most cathelicidin peptides are amphipathic and fall into one of four structural classes: α-helical peptides, disulfide bridged β-hairpin containing peptides, disulfide bridged cyclic peptides, or linear peptides which are typically much longer than any of the afore-mentioned classes and enriched in proline, tryptophan, serine, or glycine residues^16^.

Although many cathelicidin peptides have been previously described with anti-bacterial properties, relatively few have been investigated for their antiviral capabilities. Among these, only the human cathelicidin, LL-37, has received significant characterization of its antiviral activity and mechanism of action. Early experiments with LL-37 and murine CRAMP cathelicidin peptides observed inhibition of multiple influenza A virus (IAV) strains both in cell culture and in murine infection experiments^17^. Later experiments with IAV, respiratory syncytia virus (RSV), and infectious bronchitis virus (IBV) indicated that LL-37 directly affects IAV, RSV, and IBV virions during cellular infection, as LL-37 added at the time of infection effectively inhibited either virus, but not when added to cells pre- or post-infection^18–20^. The lack of inhibition observed in pre-treated cells suggests that a minority of the inhibition is dependent on conditioning of host cells and that the majority of LL-37 is consumed within a few hours of addition. The exact mechanism of direct antiviral activity seems to be both virus and model dependent. In IAV and RSV, LL-37 produced notable disruption of virions under electron microscopy^19,21^. For IBV, no virion disruption was observed by EM and the inhibitory effect was cell-type specific, suggesting an effect on other components of viral entry, such as the viral spike or host entry receptor^18^.

While cathelicidin peptides are classically depicted as relying on their amphipathicity to insert into and disrupt microbial membranes, they also stimulate an anti-microbial environment via host cell modulation and signaling. Exogenous LL-37 administration has been noted to modify the metabolic activity of host cells, their propensity for apoptosis, and their cytokine and cytokine receptor expression^22–25^. Further, cathelicidin peptides may have augmented activity *in vivo* as they exert direct chemokine-like effects to recruit immune cells to the site of infection^26,27^.

For all cathelicidins, the dichotomy of the highly conserved cathelin domain and the diverse antimicrobial domain facilitates the easy identification of putative cathelicidin peptides even from previously unexplored species. In the present study, we first identified and synthesized putative cathelicidin peptides from 16 diverse species, including bats, pangolins, humans, beluga whales, snakes, wild boar, cats, koalas, and wallabies. We then tested each of these peptides for their ability to prevent live SARS-CoV-2 from infecting permissive cells in vitro. Our screening identified a single cathelicidin peptide from the wild boar species, *Sus scrofa*, which possesses significant SARS-CoV-2 inhibitory activity in vitro. Mutational analysis and further screening of this peptide helped us create the lead-candidate peptide, Yongshi, which effectively discriminated host cells and viral particles while maintaining inhibitory activity across the alpha (B.1.1.7), beta (B.1.351), gamma (B.1.1.28.1/P.1), delta (B.1.617.2), kappa (B.1.617.1), and omicron (XBB.1.16) variants of SARS-CoV-2. We present additional in silico and in vitro binding studies that suggest Yongshi could act as a viral entry inhibitor by interfering with the SARS-CoV-2 spike protein heptad repeat 1 and 2 multimerization.

## Results

### A cathelicidin peptide of wild boar origin inhibits SARS-CoV-2

To generate the cathelicidin peptide library, we first identified previously characterized cathelicidin genes from the genomes of other species listed on The Universal Protein Resource (UniProt) and then identified additional putative cathelicidins based on homology using BLAST (Supplementary Table 1)^28^. The cathelicidin peptide was isolated and produced by solid-phase synthesis as a pure peptide for each cathelicidin gene.

**Figure 1:**
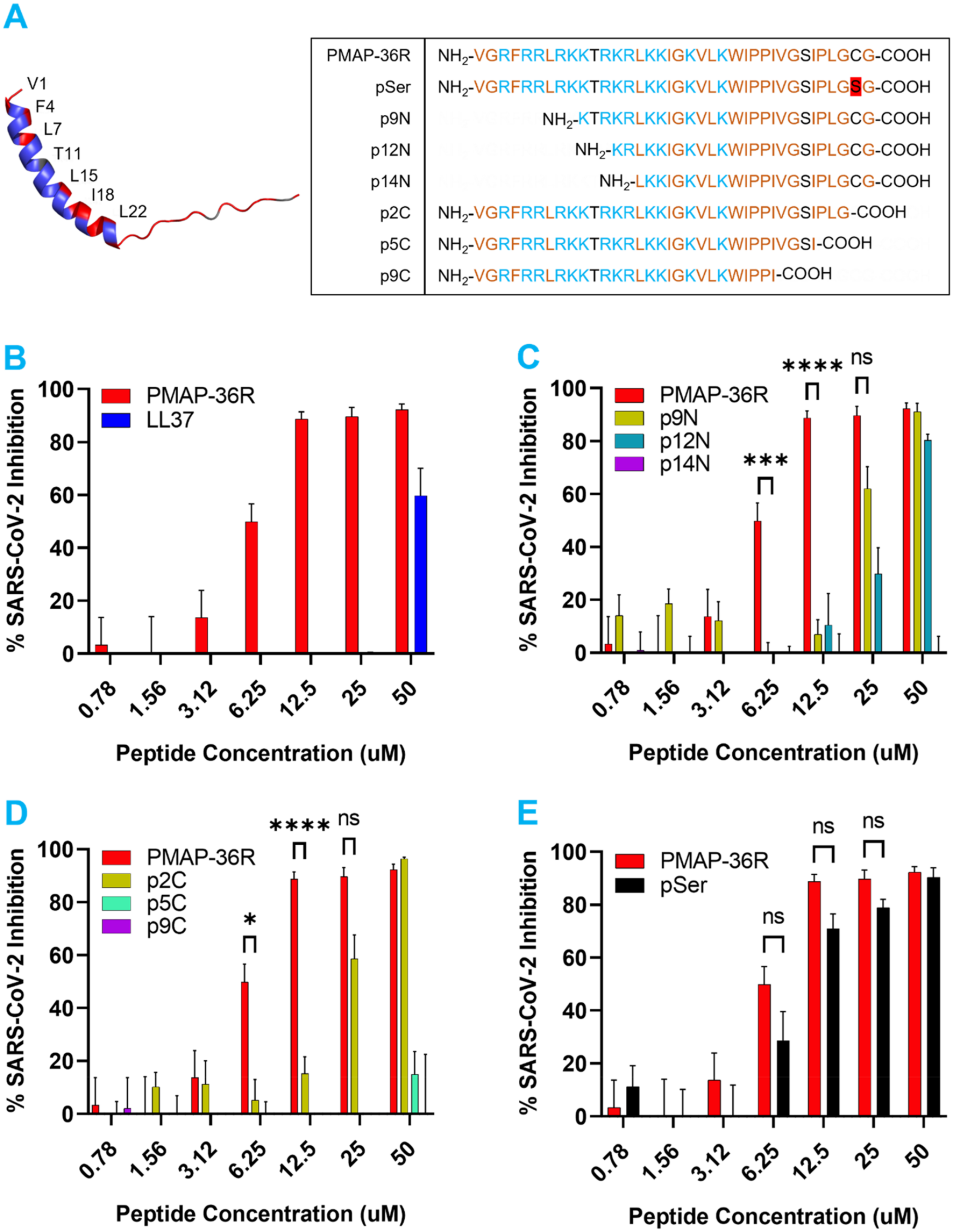
A wild boar cathelicidin PMAP-36R inhibits SARS-CoV-2 infection of Vero hACE2 cells. (**A**) 3-dimensional structure projection of PMAP-36R (left) and schematic of PMAP-36R variants (right). A 3D structure of PMAP-36R was determined by AlphaFold (P49931) and rendered in PyMOL^29,30^. Positively charged residues are depicted in blue while hydrophobic residues are depicted in red. Labeled residues along the N-terminal α-helix form a non-polar sector including the weakly hydrophilic threonine. (**B**–**E**) Inhibition of SARS-CoV-2 infection by PMAP-36R and its derivatives. PMAP-36R was compared against LL-37 (**B**), N-terminal truncations (**C**), C-terminal truncations (**D**), or a cysteine mutant (**E**) of PMAP-36R. Peptides at the labeled concentrations were pre-incubated with 100pfu of live SARS-CoV-2 virus (nCoV/USA_WA1/2020) for 1 h at 37 °C before addition to confluent Vero hACE2 cells in a 96-well plate. Infected cells were fixed and quantified by focus forming assay after 48 h. Inhibition of viral infection was calculated based on the percent area of each well staining positively for viral spike protein compared to control wells without peptide inhibitor treatment. Results are representative of 3 independent experiments performed in triplicate. Significance calculated by two-way ANOVA with Bonferonni’s correction comparing peptide derivatives against control PMAP-36R (* < .05, ** < .01, ** < .001, * < .0001).

For the initial library screening, we evaluated the inhibitory potential of each cathelicidin peptide at 50 µM by an in vitro focus forming assay (Supplemental Figure 1) or virus infectivity assay on Vero E6 cells. Using a 50% inhibitory cutoff, our initial assay identified SARS-CoV-2 inhibitory activity in 9 out of 50 candidates in the cathelicidin peptide library including LL-37 and PMAP-36R. We then tested each 50% inhibitory candidate for activity at concentrations ranging from 50 to 0.78 µM using Vero E6 cells overexpressing human ACE2 (Vero hACE2) (Fig. 1 and Supplemental Figure 2). Most candidates failed to achieve > 50% inhibition below 50 µM. Despite this trend, a candidate related to the wild boar PMAP-36 cathelicidin (PMAP-36R) maintained substantial activity even when diluted, with a calculated IC_50_ of 7.31 µM (Fig. 1B).

### Mutation analysis of PMAP-36R identifies key residues for SARS-CoV-2 inhibition

To determine the critical residues in PMAP-36R responsible for its high anti-SARS-CoV-2 activity, we synthesized and tested truncated variants of the parent cathelicidin peptide lacking residues from the N or C termini, as well as a variant without the cysteine residue (Fig. 1A). N-terminal truncates lacking 9, 12, or 14 residues (p9N, p12N, and p14N) displayed a progressive loss of anti-SARS-CoV-2 activity, with no inhibition present in p14N (Fig. 1C). By comparison, the C-terminus was more sensitive to truncation, as loss of the last two residues in p2C immediately impaired function. At the same time, little inhibitory activity remained in p5C and none in p9C (Fig. 1D). The activity of these mutants suggested that viral restriction depended on both N and C terminal amino acids (summarized in Table 1).

**Table 1:** Calculated IC_50_ and TD_50_ values for PMAP-36R derivatives and control LL-37 or OVA peptides.

	SARS-CoV-2 IC_50_ (µM)	RBC Hemolysis TD_50_ (µM)	Vero TD_50_ 48 h (µM)	HEK293T TD_50_ (µM)	Vero TD_50_ 24 h (µM)
LL-37	49.27	30.53	39.44	26.61	NA
OVA	NA	> 50	> 50	> 50	> 400
PMAP-36R	7.31	> 50	23.47	24.61	NA
Yongshi (pSer)	11.24	> 50	> 50	26.98	50.41
p9N	23.73	NA	NA	NA	NA
p12N	25.2	NA	NA	NA	NA
p14N	> 50	NA	NA	NA	NA
p2C	22.68	NA	NA	NA	NA
p5C	> 50	NA	NA	NA	NA
p9C	> 50	NA	NA	NA	NA

IC_50_ and TD_50_ values were calculated for each peptide by S-curve regression.

Interestingly, the final two residues of PMAP-36R on the C-terminus include a singular cysteine, which might be responsible for initiating covalent bonds with other PMAP-36R monomers or part of the SARS-CoV-2 virion. We also made an additional mutant of PMAP-36R by changing the penultimate cysteine to a structurally similar but non-reactive serine residue. The replacement of C36S in pSer had no significant effect on SARS-CoV-2 inhibition compared to PMAP-36R (Fig. 1E).

### pSer improves the viral specificity of PMAP-36R by reducing cytotoxicity

**Figure 2:**
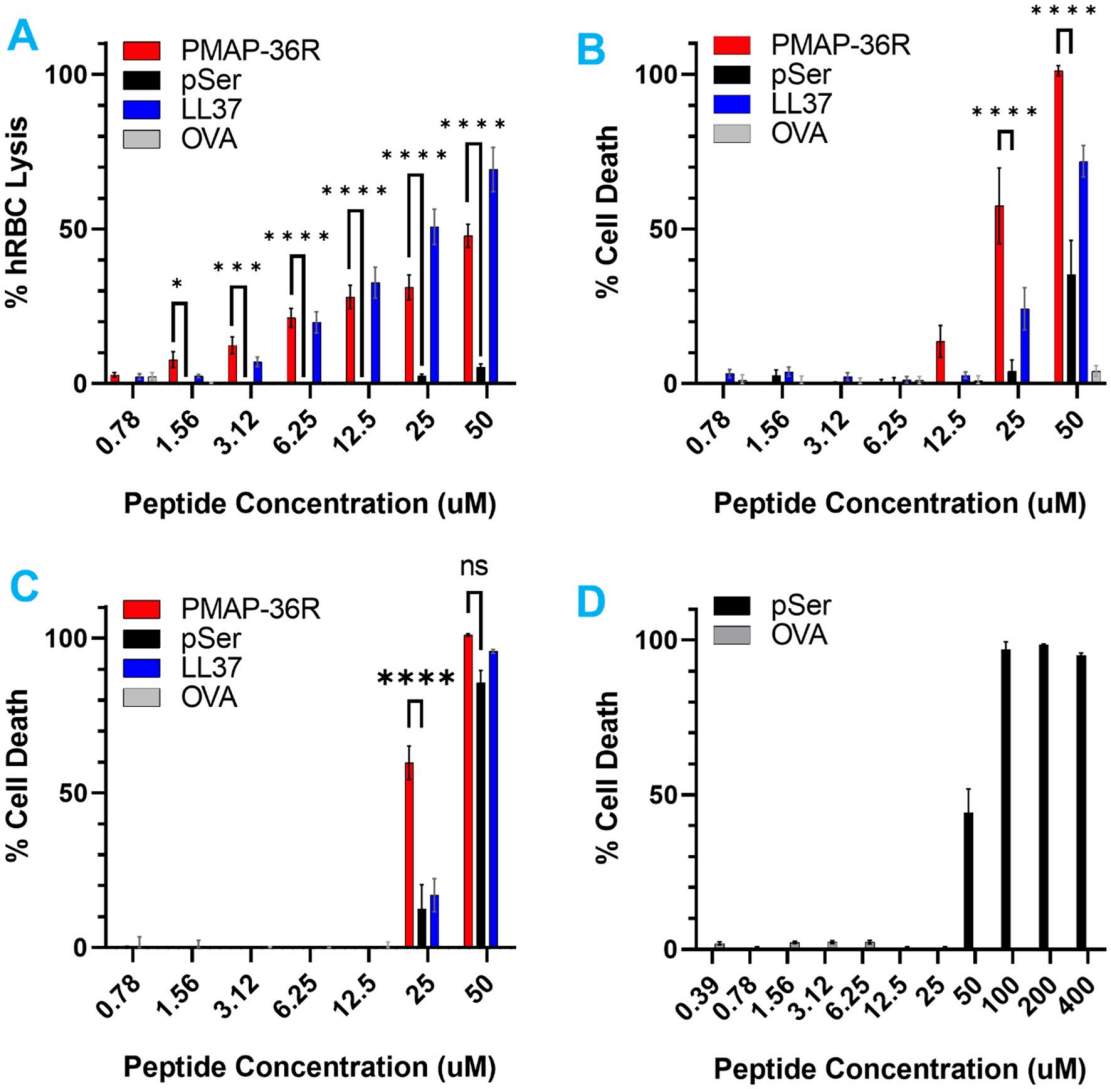
The PMAP-36R cathelicidin derivative pSer possesses reduced cytotoxicity. (**A**) Human RBC hemolysis assay showing reduced lytic activity of pSer compared to PMAP-36R or LL-37. Peptides at the labeled concentrations were incubated with human RBCs for 1 h at 37 °C in 1X PBS buffer. RBC lysis was determined by the spectral absorbance (490 nm) of cell supernatants relative to PBS-only and TritonX-100 containing controls. (**B-C**) MTS formazan formation assay with (**B**) Vero hACE2, and (**C**) HEK-293 T cell lines showing reduced cell death in wells receiving pSer compared to PMAP36-R. Peptides at the labeled concentrations were incubated with cells in 1% FBS containing DMEM for 48 h prior to the addition of MTS substrate. The percentage of cell death was calculated relative to untreated and NP-40 receiving control wells. Results are representative of 3 independent experiments performed in triplicate. (**D**) MTS formazan formation assay with the Vero A2T2 cell line showing saturation of toxic effect at 100 µM. Peptides at the labeled concentrations were incubated with cells in 1% FBS containing DMEM for 24 h prior to the addition of MTS substrate. The percentage of cell death was calculated relative to untreated and NP-40 receiving control wells. Results are representative of 2 independent experiments performed in duplicate. Significance calculated by two-way ANOVA with Bonferonni’s correction comparing pSer against control PMAP-36R (* < .05, ** < .01, ** < .001, * < .0001).

Host defense peptides, while exhibiting antimicrobial activity, often exhibit toxicity to mammalian cells. To characterize the potential of PMAP-36R for therapeutic applications, we evaluated its toxicity toward mammalian cells via a hemolysis assay with human red blood cells (RBCs). Of the peptides we tested, LL-37 and PMAP-36R had considerable hemolytic activity. Interestingly, the C36S mutant pSer exhibited significantly reduced hemolytic activity, lysing less than 10% of human RBCs even at 50 µM (Fig. 2A).

In addition to the hemolysis assay, which evaluates the immediate lytic effects, each peptide was evaluated for cytotoxicity after 48 hours of cell culture. Cytotoxicity was tested against Vero hACE2 and HEK293T cells to identify general and cell line-specific sensitivities to cathelicidin peptide treatment. We quantified cell viability by a formazan formation assay. As in the hemolysis results, pSer exhibited decreased cytotoxicity compared to the parent PMAP-36R and LL-37 (Fig. 2B,C). The toxicity from each peptide appeared independent of cell type, producing similar trends in all tested cell lines. In contrast to the hemolysis assay, PMAP-36R has greater toxicity than LL-37 when administered over 48 hours, suggesting that it may act via a slower mechanism than LL37 or differentially target the membranes of RBCs and adherent cell lines. As the toxicity of pSer did not reach saturation at 50 µM, an additional assay was performed with Vero cells evaluated at a 24-hour timepoint (Fig. 2D). These conditions did not substantially alter the observed cytotoxicity compared to Vero cells evaluated at 48 hours. As expected, pSer reached saturation around 100 µM with a calculated TD_50_ of 50.41 µM (Table 1).

The therapeutic index (TI) is a measure of safety for therapeutic drugs and, in this context, is the ratio of a compound's IC_50_ to its TD_50_. In this case, TI represents the capacity of a cathelicidin peptide to discriminate between SARS-CoV-2 virions and Vero-E6 cell membranes. LL-37 demonstrated no therapeutic potential, with a TI of < 1, as neutralization consistently lagged toxicity. The TI was slightly improved for PMAP-36R at 3.21, indicating a modest targeting of SARS-CoV-2 over Vero E6 cells. However, pSer displayed heightened specificity for SARS-CoV-2 with a TI of 4.48. We named this peptide derivative “Yongshi”, after the Mandarin word for warrior.

### Yongshi is active primarily during and after virus infection

**Figure 3:**
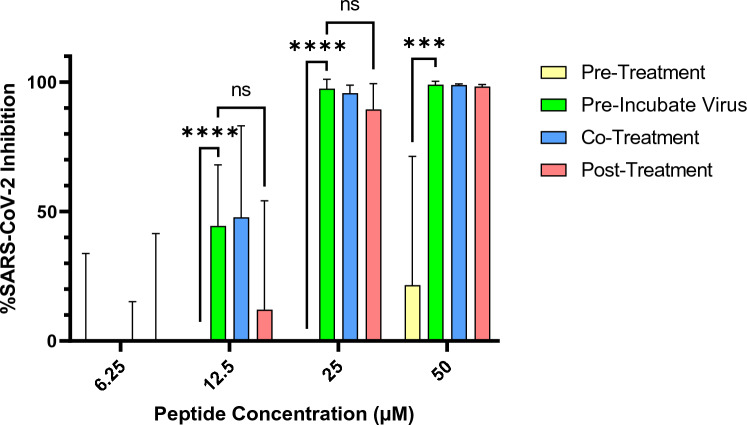
The PMAP-36R derivative Yongshi (pSer) mediates SARS-CoV-2 inhibition via acting on both virions and cells. Inhibition of SARS-CoV-2 infection of Vero A2T2 cells. Peptides at the labeled concentrations were pre-incubated with virus for 1 h or added directly to cells 1 h before, at the time of, or 1 h after infection with 100pfu of SARS-CoV-2 (nCoV/USA_WA1/2020). For pre-treatment groups, the added peptide was removed immediately prior to infection and replaced with control medium. Infected cells were fixed and quantified by focus forming assay after 48 h. Inhibition of viral infection was calculated based on the percent area of each well staining positively for viral spike protein compared to control wells without peptide inhibitor treatment. Results are representative of 2 independent experiments performed in triplicate. Significance calculated by two-way ANOVA with Bonferonni’s correction comparing each group against pre-incubated virus (* < .05, ** < .01, ** < .001, * < .0001).

To assess whether the inhibitory effects of Yongshi are mediated by cellular conditioning or direct viral neutralization, we compared the efficacy of viral inhibition when Vero hACE2 cells were treated with Yongshi 1-hour prior to, at the time of, or 1 hour after infection with SARS-CoV-2 and compared these results against cells infected with SARS-CoV-2 that was pre-incubated with Yongshi for 1 hour (Fig. 3). Pre-treatment of cells with Yongshi did not substantially inhibit SARS-CoV-2 infection at any concentration, suggesting Yongshi does not induce a significant antiviral state in host cells as has been described for LL-37^20^. By comparison, neither addition of Yongshi at the time of infection nor 1 hour following infection significantly altered viral inhibition compared to virus pre-incubated with Yongshi. These results further suggest that the inhibition by Yongshi is not dependent on the extended pre-incubation with virus but occurs rapidly at the time of infection.

### Yongshi requires chirality for its virus-inhibitory activity

**Figure 4:**
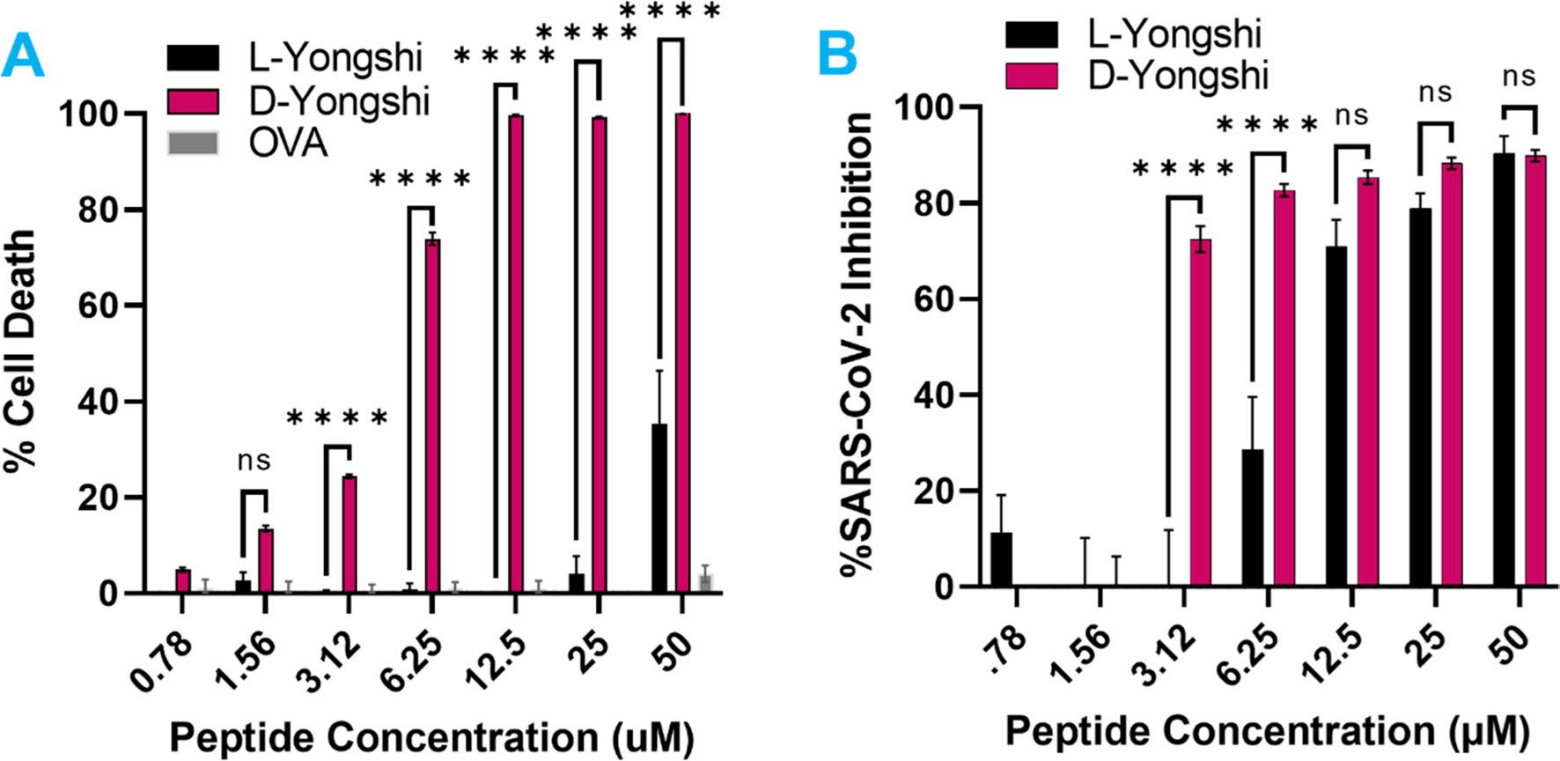
The D-enantiomer of Yongshi loses SARS-CoV-2 specificity. (**A**) Toxicity towards Vero hACE2 cells was evaluated by MTS formazan formation assay. Peptides at the labeled concentrations were incubated with cells in 1% FBS containing DMEM for 48 h prior to the addition of MTS substrate. The percentage of cell death was calculated relative to untreated and NP-40 receiving control wells. (**B**) Inhibition of SARS-CoV-2 infection of Vero hACE2 cells. Peptides at the labeled concentrations were pre-incubated with 100pfu of live SARS-CoV-2 virus (nCoV/USA_WA1/2020) for 1 h at 37 °C before addition to confluent Vero hACE2 cells in a 96-well plate. Infected cells were fixed and quantified by focus forming assay after 48 h. Inhibition of viral infection was calculated based on the percent area of each well staining positively for viral spike protein compared to control wells without peptide inhibitor treatment. Results are representative of 3 independent experiments performed in triplicate. Data for L-Yongshi reproduced from Figs. 1 and 2. Significance calculated by two-way ANOVA with Bonferonni’s correction comparing D-Yongshi against control L-Yongshi (* < .05, ** < .01, ** < .001, * < .0001).

Next, we tested whether peptide chirality had an impact on the effectiveness of Yongshi antiviral activity. To do this, we synthesized a stereoisomer or mirror image variant of Yongshi using D rather than L amino acids. The resulting peptide, D-Yongshi, has an identical sequence and amphipathicity but a mirrored 3D conformation, permitting the dissection of Yongshi's inhibitory activity into sequence-based and structure-based components. Compared to the L-Yongshi peptide, D-Yongshi possessed greatly increased cytotoxicity (Fig. 4A). Although we tested D-Yongshi against SARS-CoV-2, viral inhibition was only observed at concentrations that also induced substantial cell death (Fig. 4B). The apparent inability of D-Yongshi to discriminate between SARS-CoV-2 and host cells supports the existence of specific interactions between L-Yongshi and Vero cells or SARS-CoV-2, dictated by secondary and tertiary structure.

### Yongshi inhibits emerging SARS-CoV-2 variants of concern

**Figure 5:**
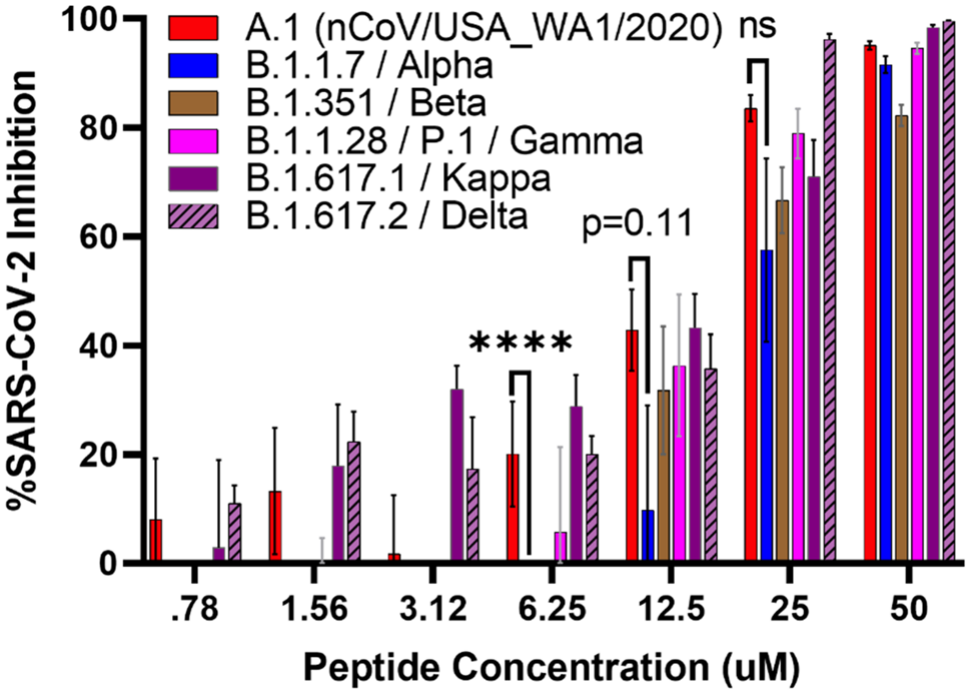
Yongshi retains inhibitory activity against emergent SARS-CoV-2 variants alpha, beta, gamma, kappa, and delta. Dilutions of the Yongshi peptide were tested for inhibition of SARS-CoV-2 and its drifted variants. Peptides at the labeled concentrations were pre-incubated with 100pfu of the indicated SARS-CoV-2 variant virus for 1 h at 37 °C before addition to confluent Vero hACE2 cells in a 96-well plate. Infected cells were fixed and quantified by focus forming assay after 48 h. Inhibition of viral infection was calculated based on the percent area of each well staining positively for viral spike protein compared to control wells without peptide inhibitor treatment. Results are representative of 2 to 3 independent experiments performed in triplicate. Significance calculated by two-way ANOVA with Bonferonni’s correction comparing A.1 inhibition against each variant (* < .05, ** < .01, ** < .001, * < .0001).

Since its emergence in late 2019, SARS-CoV-2 has continued to change: multiple new variants of concern have emerged, defined primarily by acquired mutations in the spike protein. Mutations to the receptor-binding domain, such as, L452R, E484K and N501Y have garnered particular concern as they threaten to erode the protection provided by the original A.1 isolate-based vaccines and monoclonal antibody therapies^31^. Since we identified Yongshi by screening peptides against the A.1 isolate of SARS-CoV-2, we sought to determine the extent to which Yongshi would inhibit drifted variants of SARS-CoV-2. We, therefore, tested Yongshi against five variants of concern: B.1.1.7 (alpha), B.1.351 (beta), B.1.1.28.1/P.1 (gamma), B.1.617.2 (delta), and one newly emergent variant of interest B.1.617.1 (kappa). The defining mutations of each variant are mostly concentrated in the spike S1 region responsible for ACE2 binding. The mutations in the spike protein for these variants are as follows. Alpha (69-70del, 144del, E484K, S494P, N501Y, A570D, D614G, P681H, T716I, S982A, D1118H), Beta (D80A, D215G, 241-243del, K417N, E484K, N501Y, D614G, A701V), Gamma (L18F, T20N, P26S, D138Y, R190S, K417T, E484K, N501Y, D614G, H655Y, T1027I), Kappa (T95I, G142D, E154K, L452R, E484Q, D614G, P681R, Q1071H), and Delta (T19R, V70F, T95I, G142D, E156-, F157-, R158G, A222V, W258L, K417N, L452R, T478K, D614G, P681R, D950N). Not surprisingly, the spike S2 region responsible for viral fusion is heavily conserved, especially regions corresponding to the fusion peptide and heptad repeats. The fusion peptide is conserved across all five variants, and only the delta variant possesses a mutation in a heptad repeat (D950N)^32^. We tested Yongshi against these variant viruses and our data, shown in Fig. 5, demonstrate that as expected, Yongshi inhibited all the variants tested, albeit with reduced activity against the Alpha variant amounting to a less than 2-fold decrease in IC_50_ (Table 2). The varied sensitivity of each variant to Yongshi may reflect differences in the efficiency of membrane fusion. An increased propensity for variant fusion at the plasma membrane may underlie the observed reduction in viral inhibition.

**Table 2:** Calculated IC_50_ values for Yongshi against drifted SARS-CoV-2 variants.

Variant	IC_50_ (µM)
A.1	12.13*
B.1.1.7 (Alpha)	20.54
B.1.351 (Beta)	21.64
B.1.1.28 (Gamma)	16.11
B.1.617.1 (Kappa)	17.34
B.1.617.2 (Delta)	13.28

IC_50_ values were calculated for each variant by S-curve regression.

*The IC_50_ of A.1 is calculated from a set of experiments performed at the same time as the other variants and therefore varies slightly from the IC_50_ presented in Table 1.

### Deep-learning sequence comparison and computational modeling predict mimicry of Yongshi to the spike HR1/HR2 regions

**Figure 6:**
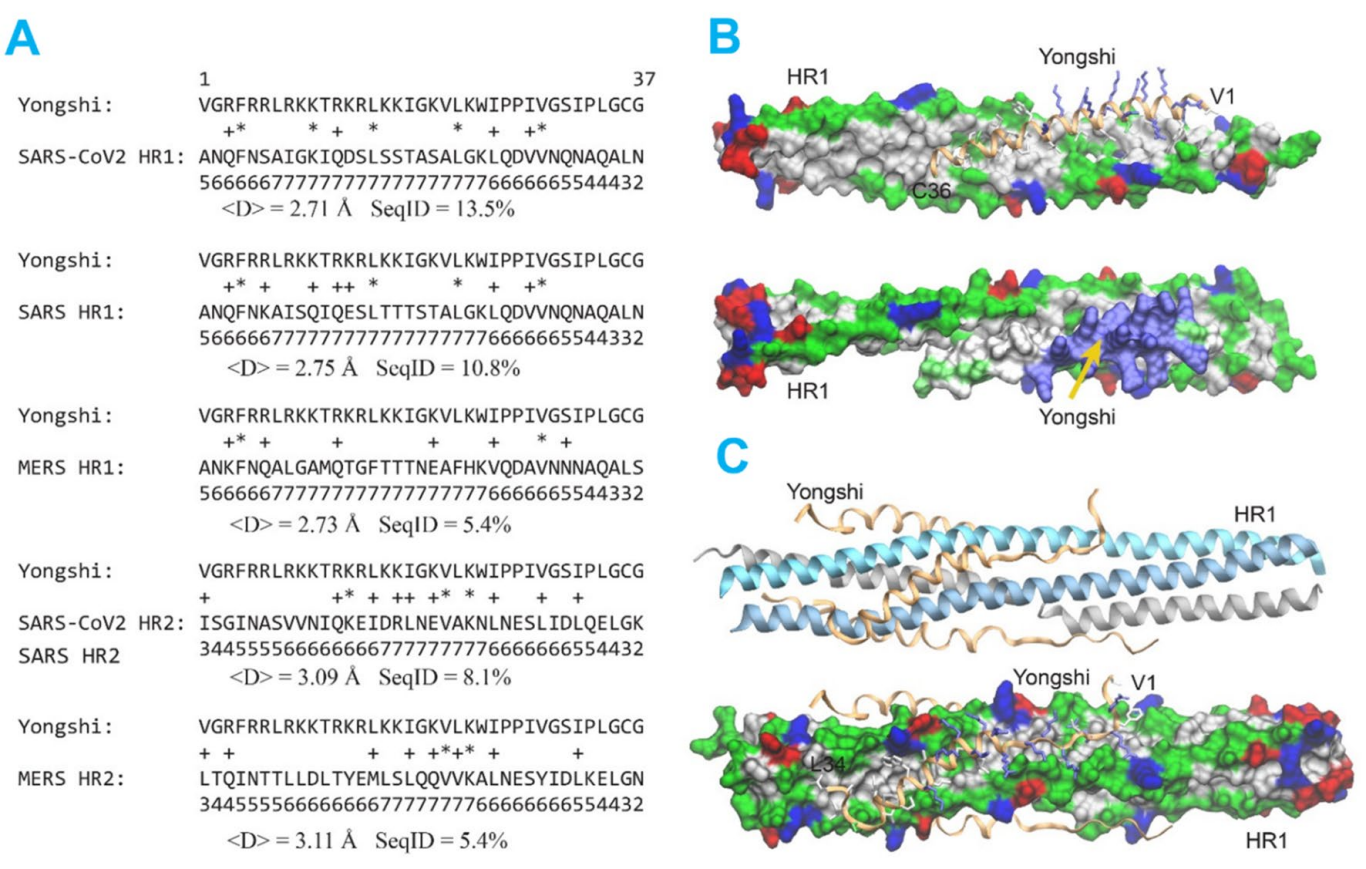
Deep-learning sequence alignment algorithm and computational modeling predict stable binding interactions between Yongshi and HR1. (**A**) Sequence alignments between Yongshi and HR1/HR2 sequences of SARS-CoV-1, SARS-CoV-2, and MERS-CoV by SAdLSA^36^. < D > denotes the mean of predicted distance between aligned residues, and SeqID is Sequence Identity. Identical and statistically favorable amino acid substitutions are denoted by “*” and “+”, respectively. The digits below each alignment indicate the probability that the predicted structure for each aligned amino acid pair will be less than 3 Å, e.g., the number 6 predicts a probability score between 60 to 70%. Note that SARS-CoV-1 and SARS-CoV-2 have identical sequences in their HR2 regions aligned to Yongshi. (**B**) A structural model of two SARS-CoV-2 HR1s in complex with a single Yongshi peptide. The two HR1s are shown in the surface representation, and the Yongshi peptide is shown in ribbon backbone and licorice sidechain representations in the upper snapshot, and the same surface representation in the lower snapshot. The lower snapshot is slightly rotated from the upper snapshot to show a large positively charged surface patch of Yongshi as pointed by a yellow arrow. The color codes for amino acids are hydrophobic (white), polar (green), positive (blue), negative (red). (**C**) A structural model of three SARS-CoV-2 HR1s in complex with three Yongshi peptides after 100 ns of MD simulation. The upper snapshot is shown in cartoon representation for three HR1s (blue, cyan, grey), and ribbon representation for Yongshi (yellow). The lower snapshot is in the same orientation as the upper one but shown in the same representation as snapshots in (**B**).

What is the molecular mechanism of Yongshi’s inhibition of the SARS-Cov-2 virus? A common mechanism of peptide inhibition is mimicry of functionally vital interactions. To find such a clue for Yongshi, we searched its sequence against a representative set of sequences whose structures (and some of their molecular interaction partners) have been determined in the Protein Data Bank (PDB)^33^. For this analysis, classic sequence search tools such as PSI-BLAST^34^ and more sensitive HHsearch^35^ yield a very small number of hits and do not find any hit related to the proteins of SARS-CoV-2. We then applied our recently developed algorithm SAdLSA^36^, a deep-learning based sequence alignment method that shows significantly improved accuracy via learning the structural fold or motif representations encoded by protein sequences^37^. When applied to a recent PDB sequence library (~ 83,000 sequences, see Methods), among the top 1% sequences ranked in comparison to Yongshi, SAdLSA identified the Heptad Repeat 1 and 2 (HR1 and HR2) of SARS Spike protein in PDB entry 1ZV8, HR2 of SARS-Cov-2 in PDB entry 6LVN, as well as HR repeats in respiratory syncytial virus (PDB 3KPE) and Mumps virus (PDB 2FYZ). Figure 6A shows SAdLSA alignments between Yongshi and HR1/2 of SARS, SARS-CoV-2, and MERS, respectively. While the HRs are disordered coiled coils in the pre-fusion stage of the coronaviruses, they form a 6-helix bundle serving as the “fusion core” as observed in the post-fusion conformation of the virus, whereby three HR1s form a central 3-helical bundle, and three HR2s bind to the central core forming the second layer of the complex^38,39^. SAdLSA alignments indicate that Yongshi could mimic either HR1 or HR2, with somewhat higher similarity to HR1 than HR2. The latter has disordered terminal regions that lead to a relatively lower similarity score to Yongshi. Most importantly, Yongshi also exhibits a single helical motif and displays a similar hydrophobic pattern, matching its counterpart in HR1 or HR2. Therefore, we hypothesized that Yongshi could inhibit the SARS-CoV-2 fusion machinery by disrupting the critical HR1/HR2 complex via binding to the HR1 domain. This hypothesis is supported by previous observations that a peptide mimetic of the HR2 of the SARS virus is effective in inhibiting the virus^40,41^.

To evaluate the possibility that Yongshi might act as an HR1 or HR2 mimetic, we built respective structural models for both scenarios using the SAdLSA alignments (Fig. 6A) and a crystal structure of HR1/2 complex of SARS-CoV-2^39^. Figure 6B shows a structural model of two HR1s of SARS-CoV-2 complexed with one Yongshi peptide, replacing the third HR1 with the aligned Yongshi peptide. After energy minimization (see Methods), the complex is stabilized with Yongshi's hydrophobic residues fitting to the complementary hydrophobic core originally formed by HR1s. Eight positively charged lysines or arginines of Yongshi point outwards and form a charged surface patch, dramatically reducing the hydrophobic groove originally reserved for interacting with HR2s and potentially disrupts the HR1/HR2 interactions. Figure 6C shows the second structural model of three HR1s of SARS-CoV-2 in complex with three Yongshi peptides, by replacing three HR2s originally found in the template. Since there are several clashes between lysines and the hydrophobic core, we performed 100 ns molecular dynamics simulations to explore the stability of this putative complex. Figure 6C shows snapshots at the end of the MD simulation. The N-termini of the Yongshi peptides appear flexible and their movements eliminate the originally unfavorable charged-hydrophobic interactions. In contrast, the hydrophobic interactions between the short helix of Yongshi and HR1s are well-maintained throughout the simulations with a root mean square deviation below 3 Å. The modeling and MD simulation suggest that Yongshi could disrupt HR1 and HR2 interactions via two possible mechanisms by mimicking the HR1 helical core and/or competing for binding to HR1, both involving direct interactions with HR1s. We also note that the D950N mutation in the delta variant as well as the Q954H and N969K mutations in the omicron variant are unlikely to impede hypothetical interactions between Yongshi and HR1 since they occur outside the binding interface.

### L-Yongshi but not D-Yongshi, binds heptad repeat 1 of SARS-CoV, MERS-CoV, and SARS-CoV-2

**Figure 7:**
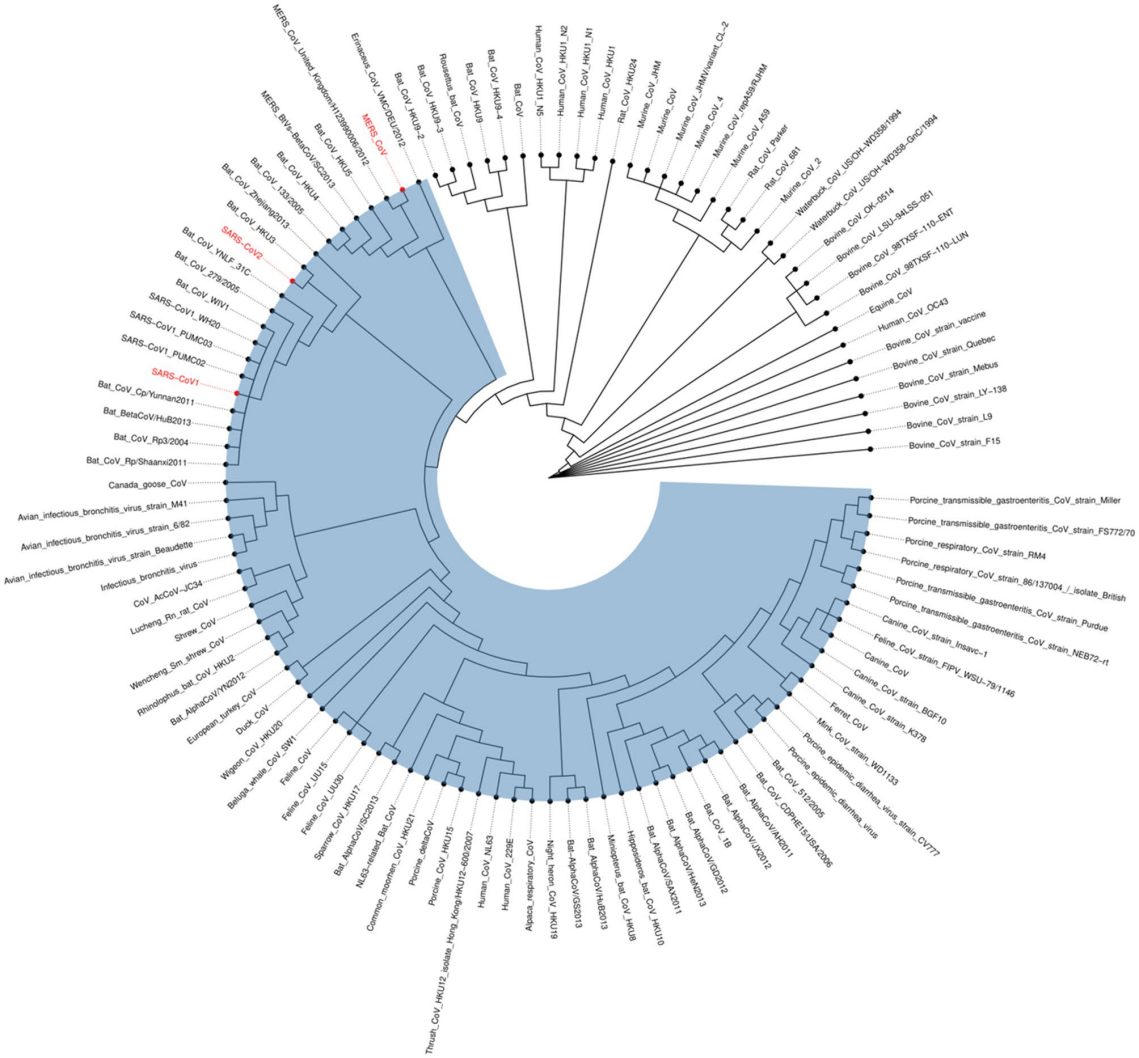
Phylogenetic tree of HR1 segments in disparate coronaviruses. 111 coronavirus spike protein HR1 sequences that share homology with the HR1 of SARS-CoV-2 were identified by BLASTp^28^. Sequences were aligned using MUSCLE and the resultant phylogenetic tree was visualized with ggtree^42,43^. Three of the HR1 sequences (red) were analyzed in detail in panel B and they share a common ancestor sequence near the root of the tree. The inferred breadth of coverage is shaded (blue). The three HR1 peptides we tested for binding with Yongshi peptide are marked in red.

**Figure 8:**
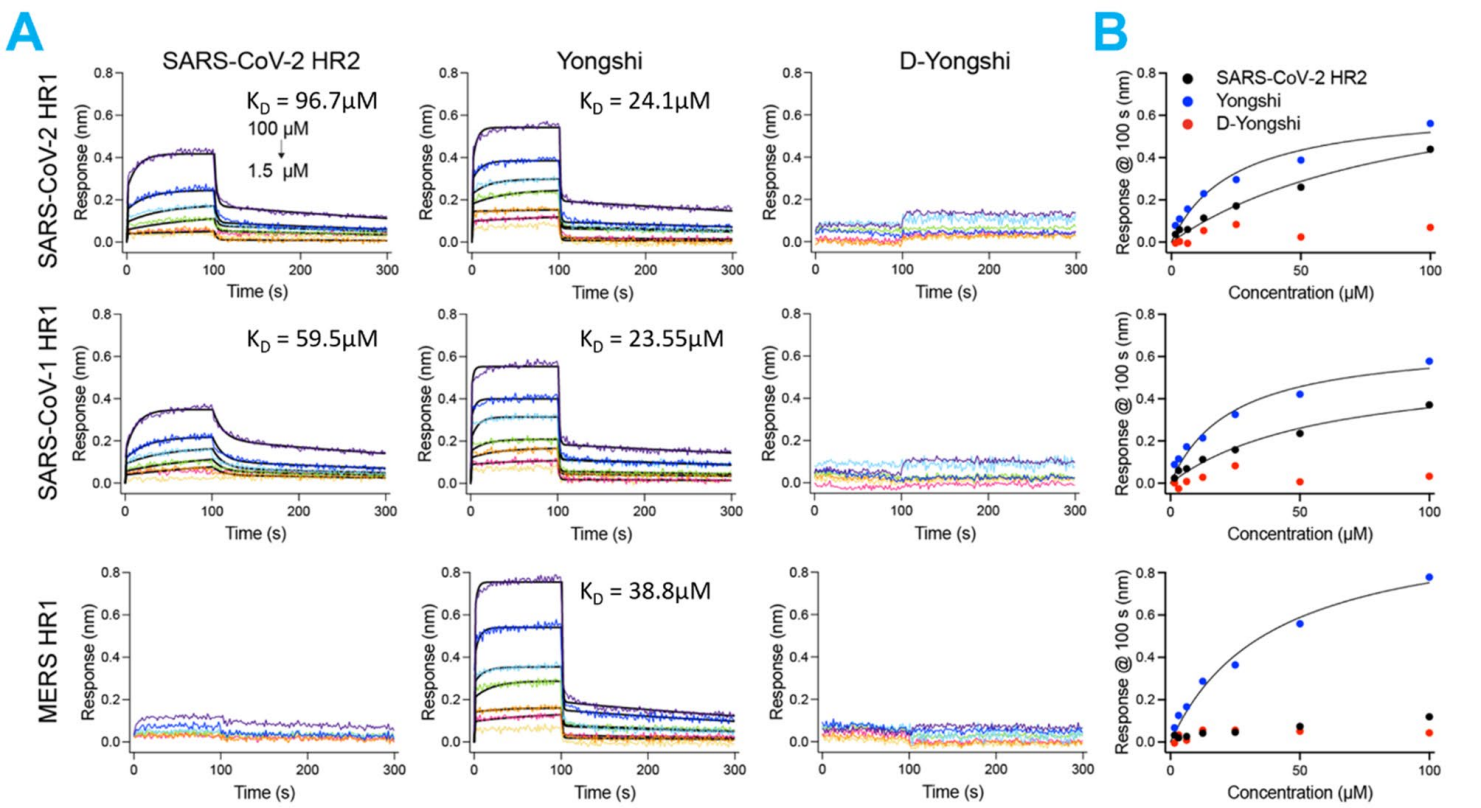
Yongshi binds to HR1 peptide with higher affinity than HR2. Biotinylated HR1 peptide from SARS-CoV-2, SARS-CoV-1, or MERS-CoV was bound to a streptavidin-coated bio-layer interferometry sensor and incubated with serial dilutions of either SARS-CoV-2 HR2, Yongshi, or D-Yongshi starting from 100 μM. (**A**) Representative plots of peptide binding over 100 s of association followed by 200 s of dissociation. (**B**) Regression analysis of the peptide binding response at each concentration after 100 s of association. Relative steady state affinities were derived from the K_D_ of the hyperbola.

While computational projections are promising, experimental analysis is critical for confirmation. Using BLASTp, we identified 111 coronavirus spike protein HR1 sequences that share homology with the HR1 of SARS-CoV-2 (Fig. 7)^28^. Out of these, we synthesized three biotinylated HR1 peptides (SARS-CoV-2 HR1, SARS-CoV-1 HR1 and MERS-CoV HR1) and tested their ability to bind SARS-CoV-2 HR2, L-Yongshi, and D-Yongshi peptides. We used bio-layer interferometry to measure the binding of immobilized HR1 peptides to serial dilutions (100–1.56 μM) of SARS-CoV-2 HR2, L-Yongshi, and D-Yongshi (Fig. 8). As expected, SARS-CoV-2 HR2 bound to the HR1 of both SARS-CoV-2 and SARS-CoV-1 with a moderate affinity of 96.7 μM and 59.5 µM respectively but showed no binding to MERS-CoV HR1. Interestingly, L-Yongshi showed dose-dependent binding to all three HR1 peptides tested (SARS-CoV-1, MERS-CoV, and SARS-CoV-2), suggesting that Yongshi could potentially act as a pan-coronavirus entry inhibitor. Among the three HR1 peptides, L-Yongshi bound with the strongest relative affinity to the HR1 of SARS-CoV-1 (23.55 μM). L-Yongshi bound to SARS-CoV-2 HR1 with an approximately 4-fold stronger affinity (24.1 μM) than SARS-CoV-2 HR2 (96.7 μM), indicating that it could potentially outcompete HR2 for binding to HR1. As a control, we tested D-Yongshi for binding to all three HR1 peptides, and the D-enantiomer did not show binding to any HR1 peptides up to 100 μM. These binding experiments suggest that Yongshi can both specifically bind to the HR1 of coronaviruses and that it could outcompete HR2 for binding to HR1. The specificity of this interaction is dependent on some secondary structure as the chirality (L- vs. D-enantiomer) is critical for binding. Thus, we posit that Yongshi could act as a viral entry inhibitor by blocking the fusion machinery of coronaviruses.

## Discussion

**Figure 9:**
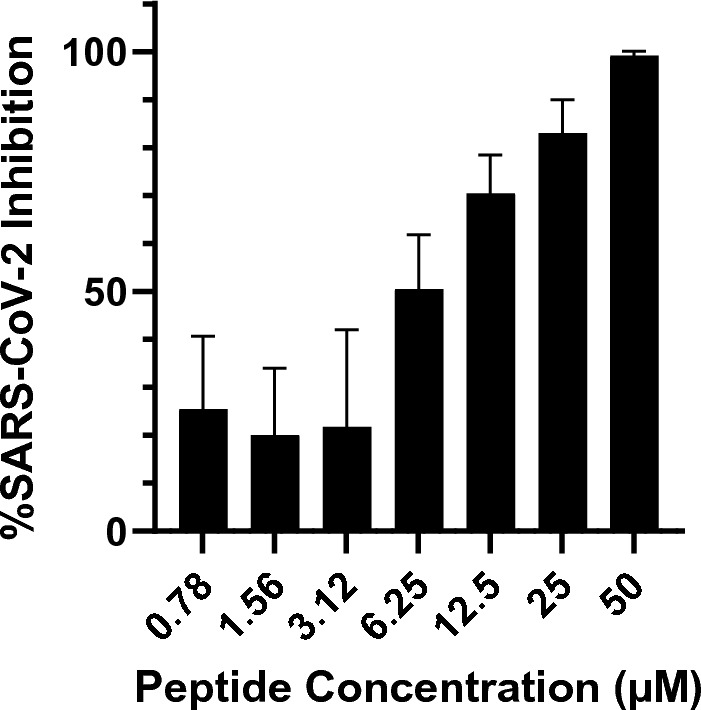
Yongshi remains active against the SARS-CoV-2 Omicron variant. Peptides at the labeled concentrations were pre-incubated with 100pfu of the SARS-CoV-2 XBB.1.16 variant virus for 1 h at 37 °C before addition to confluent Vero hACE2 cells in a 96-well plate. Infected cells were fixed and quantified by focus forming assay after 24 h. Inhibition of viral infection was calculated based on the percent area of each well staining positively for viral spike protein compared to control wells without peptide inhibitor treatment. Results are representative of 2 independent experiments performed in triplicate.

Here we identified a cathelicidin peptide of wild boar origin that inhibits SARS-CoV-2 and exhibits binding to the HR1 of SARS-CoV-2 spike protein. The specificity of this interaction is further supported by the inability of the mirror-image peptide, D-Yongshi to target SARS-CoV-2 or bind to the HR1 peptide. This interaction may contribute to the observed inhibition of SARS-CoV-2 but is likely only one of several mechanisms in addition to direct effects on the viral membrane or induction of an antiviral state in host cells. These results identify how Yongshi can act via both direct and indirect mechanisms, presenting multiple avenues for further engineering of peptide derivatives. Further, as SARS-CoV-2 has undergone significant evolution since our initial studies, we repeated our analysis with the recent XBB.1.16 variant and find that Yongshi continues to provide substantial virus inhibition on par with the original wild-type virus (Fig. 9).

For millennia, host defense peptides have co-evolved with their hosts to best protect them against pathogens in their niche. Unlike antibodies or T cell receptors which are highly diverse, and which the individual host makes > 10^7^ specificities, host defense peptides are produced in very limited numbers; for instance, humans produce a single cathelicidin LL-37. Yet these host defense peptides work because they often target an Achilles’ heel or a conserved motif that is crucial for microbial survival. These targeted motifs are often common among multiple pathogens, even those that do not infect the given species. Because of this feature, if a pathogen contains the motif or element that the peptide acts upon, the host defense peptide can cross-neutralize that bacteria or virus, regardless of the species that encounter it. It is highly unlikely that Yongshi arose to combat SARS-CoV-2 as the virus only recently emerged, but instead Yongshi confers the wild boar protection against some related virus that bears a complementary HR1 peptide. In this context, Juergen Richt and colleagues have reported that five-week-old pigs are not susceptible to infection with SARS-CoV-2 infection. Briefly, they infected pigs with 10^6^ TCID_50_ dose of SARS-CoV-2 via oral, intranasal or intratracheal route and the virus did not replicate in pigs, and they did not produce antibodies against SARS-CoV-2 either^44^. Whether this protection is due to the Yongshi-related cathelicidin, PMAP-36 remains to be investigated.

Regarding the specific mechanism of HR2 mimicry, several other purpose-built HR2 mimetics have been previously described to inhibit SARS-CoV-2. Early HR2 mimetics designed against SARS-CoV-2 used the core HR2 region (amino acids 1163-1202) with additional amino acid substitutions or peptide modifications to achieve viral IC_50_ values in the low micro-molar to high nano-molar range^39,45–48^. Recently, a crystal structure of an extended HR1HR2 6-helix bundle led to the design of an extended HR2 mimetic which achieves an impressive IC_50_ of 1.5nM against SARS-CoV-2. This activity was dependent on the expression of TMPRSS2 for receptor mediated entry and was maintained against alpha, delta, and omicron variant viruses with similar active concentrations^49^. It is not surprising that HR2 derived peptides can vastly outcompete Yongshi in performance as an HR2 mimetic, however, it is curious that Yongshi achieves a comparable IC_50_ to the unmodified, core HR2 region which varies by model system and is reported between 0.98 µM and 33.74 µM^46–48^. Given that Yongshi also aligns closely with the heptad repeats of RSV and Mumps, we hypothesize that PMAP-36R may have evolved in response to multiple endemic porcine viruses to achieve broad heptad repeat compatibility. It is unclear whether PAMP-36R is expressed at the level necessary to achieve this activity in nature, but LL-37 has been reported in humans at 20 µg/mL (~ 4.5 µM) in bronchoalveolar lavage fluid and up to 80 µg/mL (~ 17.8 µM) in nasal secretions. Notably these concentrations are attained in response to systemic and local inflammation, with healthy individuals producing significantly less peptide^50,51^.

In searching for the previous characterization of the Yongshi sequence, we identified that the Yongshi peptide is two amino-acids longer than the mature PMAP-36 cathelicidin, which lacks the N-terminal valine and undergoes a C-terminal amidation reaction which removes the terminal glycine^52^. Although PMAP-36 has not been studied in the context of viral infection, multiple publications demonstrate a bacterial inhibition curve similar to the inhibition of SARS-CoV-2 by Yongshi. In the context of *E. coli*, Scheenstra and colleagues demonstrated a minimal bactericidal concentration for PMAP-36 in the range of 5–10 µM. Further, removing up to 11 N-terminal residues or mutation of cysteine to serine did not reduce *E. coli* killing potential but reduced cell toxicity as measured by both porcine RBC hemolysis and RAW264.7 cell mitochondrial activity in culture^53^. While our model describes a specific interaction between Yongshi and SARS-CoV-2, the parallel effects of mutagenesis against SARS-CoV-2 and *E. coli* suggest the mechanism of action has shared components whether the target is viral or bacterial. Alternatively, transmission electron micrographs of PMAP-36 treated *E. coli* document vesicle shedding, a bacterial stress response to lipid asymmetry^53,54^. Combined with our results, this observation may indicate that SARS-CoV-2 is susceptible to lipid asymmetries caused by Yongshi, but not the amphipathic mechanisms of other cathelicidins^55^.

L-Yongshi, but not D-Yongshi, inhibits SARS-CoV-2 and its drifted variants. Amino acids can be produced as enantiomers, which are mirror images of each other. Of these two configurations, the L-enantiomer, in contrast to the D-enantiomer, is almost exclusively produced naturally. Because of this, host proteases, for the most part, only demonstrate enzymatic activity against the L-enantiomer of peptides and proteins. In contrast, D-enantiomer peptides and proteins are not susceptible to this natural degradation. However, since only the L-Yongshi demonstrated antiviral activity, we must formulate L-Yongshi appropriately, for therapeutic or prophylactic evaluation in vivo. While Yongshi possesses some inhibitory activity, further modifications to the peptide are necessary to generate a therapeutically viable product. Given the existence of purpose built HR2 mimetics with much lower IC_50_ ranges than Yongshi, future designs should further explore the cellular effects of Yongshi or the ability to differentially disrupt viral and host membranes. Additionally, we note that administering Yongshi as a single bolus is sub-optimal in that it increases immediate cytotoxicity and has a short half-life for cellular protection. A better approach may be to design Yongshi as a synthetic cathelicidin to be delivered by an mRNA vector and thereby produced locally in situ.

## Methods

### Identification of zoonotic cathelicidins

Cathelicidin peptide sequences were identified using the UniProt database and analogous peptide sequences were identified using the nBLAST databases. Sequences were analyzed for homology to the antimicrobial domain of Human Cathelicidin LL-37, as variation from LL-37 was preferred. Putative peptide sequences were modeled using I-TASSER for antimicrobial peptide-like secondary structural features, analyzed for net charge, and then searched for preexisting characterization^56^. Sequences with 11–50 amino acids that were not previously described for antiviral activity were chosen as candidates.

### Synthesis of cathelicidin peptides and SARS-CoV-2 peptides

Unlabeled and biotinylated zoonotic cathelicidin peptides and SARS-CoV-2 peptides were produced by standard Fmoc synthesis by Genemed Synthesis Incorporated. Peptides were purified to > 95% purity by HPLC as confirmed by mass-spectroscopy. Recombinant human LL-37 was purchased from Anaspec (Catalogue#: AS-61302). Recombinant OVA peptide was purchased from Invivogen (Catalogue#: vac-sin). For viral inhibition and cell toxicity assays, lyophilized peptides were first reconstituted in DMSO to a concentration of 10 mM before further dilution in DMEM or PBS.

### Cell lines and growth conditions

Vero E6 (ATCC CRL-1586) and HEK293T (ATCC CRL-1573) were purchased from ATCC. Cells were maintained at 5% CO_2_ and 37 °C in a humidity controlled incubator (Forma Series II model 3110) in Dulbecco’s modified eagle medium (DMEM, FisherScientific, Catalogue#: 12-614Q) supplemented with 10% heat inactivated fetal bovine serum (FBS, Rockland, Catalogue#: FBS-02-500), 2 mM L-glutamine (Quality Biological, Catalogue#: 118-084-721) and 1X concentrations of penicillin, streptomycin, and amphotericin B (P/S/A, Quality Biological, Catalogue#:120-096-711).

### SARS-CoV-2 strains and growth conditions

The following previously described isolates were used for each SARS-CoV-2 variant: A.1 (nCoV/USA_WA1/2020), B.1.1.7/Alpha (SARS-CoV-2/human/USA/CA_CDC_5574/2020), B.1.351/Beta (hCoV-19/South Africa/KRISP-k005325/2020), B.1.1.28/P.1/Gamma (hCoV-19/Japan/TY7-503/2021), B.1.617.1/Kappa (hCoV-19/USA/CA-Stanford-15_S02/2021), B.1.617.2/Delta (hCoV-19/USA/PHC658/2021), XBB.1.16/Omicron (hCoV-19/USA/CA-Stanford-139_S23/2023)^57–59^. Viruses were passaged on Vero ACE-2 or Vero-TMPRSS2 cells and subjected to next-generation sequencing to confirm identity. Viral stocks were stored at -80֯°C and titered by focus forming assay on Vero E6 cells.

### Data visualization and analysis

Data visualization and analysis for SARS-CoV-2 peptide inhibition, human RBC hemolysis toxicity, and cell viability assays was performed using GraphPad Prism 9.2.0. Outliers were defined as greater than 1.5 the interquartile range above or below the boundary of the interquartile range and excluded from analysis. Graphical axes are cut at 0% and 100%, data points beyond this range are not visualized but are included in the calculation of the mean and standard error. IC_50_ and TD_50_ values were calculated by S-curve regression of the plotted data.

### SARS-CoV-2 peptide inhibition assay

2 × 10^4^ Vero E6 cells were seeded to each well of a poly-L lysine coated 96-well tissue culture plate (Corning, Catalogue#: 354516) and allowed to adhere overnight. The following day, SARS-CoV-2 virus was diluted in DMEM without FBS or P/S/A such that 50 µL contains 100 pfu of virus. 50 µL of virus in DMEM was then combined with 50µL of peptide dilution in DMEM containing 2% FBS and 2X P/S/A and incubated at 37 °C and 5% CO_2_ for 1 h. Media was then aspirated from Vero E6 cultures and replaced with 100µL of virus-containing media per well. Plates were then incubated at 37 °C and 5% CO_2_ for an additional 48 h. Cells were fixed by removal of virus media and incubation with 2% paraformaldehyde in PBS. Cells were permeabilized by 1X PBS + 0.1% saponin (Sigma, Catalogue#: 47036-50G-F) and 0.1% BSA (Sigma, Catalogue#: A2153-500G). Cross-reactive SARS-CoV-1 clone CR3022 monoclonal antibody (Abcam, Catalogue#: ab273073-200ug) was used to detect SARS-CoV-2 spike protein expression in combination with Goat anti-human IgG-HRP secondary antibody (SouthernBiotech, Catalogue#: 2045-05). Virus infected cells were visualized by adding TruBlue Peroxidase Substrate (SeraCare, Catalogue#: 50-78-02) and the plates were scanned and the spots counted using a custom Matlab script we developed in house. All infection assays were executed in a BSL-3 lab environment following appropriate handling and disposal guidelines.

### Hemolysis toxicity assay

Washed Red Blood Cells (RBCs) were purchased from (Innovative Research Inc, Catalogue#: IWB3ALS40ML). 1 mL of RBCs was washed once in cold 1X PBS and then resuspended in 20 mL of cold 1X PBS. In a 96-well format, 10 µL of RBCs was added to each well containing 90 µL of either peptide pre-diluted in 1X PBS, 1X PBS only, or 1X PBS + 0.1% Triton-X100 (Fisher Scientific, Catalogue#: BP151-100). Cells were briefly mixed on an orbital shaker then incubated at 37 °C for 1 h with 5% CO_2_. After incubation, cells were pelleted in a benchtop centrifuge at 500 rcf for 3 min and supernatants were collected for reading at 490 nm on a Biotek Synergy 2 Multi-Mode Microplate Reader. The percentage of RBC lysis was calculated by linear regression using the PBS and PBS + 0.1% TritonX-100 as 0% and 100% lysis controls, respectively. Statistical significance was calculated by two-way ANOVA with Bonferroni’s correction.

### Cell viability assay

2 × 10^4^ Vero E6 or HEK293T cells were seeded to each well of a 96-well tissue culture plate (CellTreat, Catalogue#: 229197) and allowed to adhere overnight. 24 h later, cell culture media was aspirated and replaced with 100 µL peptide containing or control DMEM with 1% FBS and 1X P/S/A. Cells were then incubated at 37 °C and 5% CO_2_ for 48 h. After incubation, 0% viability control wells received 1µL of NP-40 (Sigma-Aldrich, Catalogue#: I3021-50ML). 5 min after the addition of NP-40, 20 µL of CellTiter 96-Aqueous One (Promega, Catalogue#: PAG3580) was added to each well and briefly mixed on an orbital shaker. Cells were incubated for 1 h at 37 °C and 5% CO_2_. Plates were briefly shaken to mix formazan products before the transfer of supernatants to a clear bottom 96-well assay plate (Costar, Catalogue#: 3912). Formazan concentrations were measured at 490 nm on a Biotek Synergy 2 Multi-Mode Microplate Reader. The percentage of viable cells was calculated by linear regression using DMEM-only and DMEM + 1% NP-40 as 100% and 0% cell viability controls, respectively. Statistical significance was calculated by two-way ANOVA with Bonferroni’s correction.

### Modeling of Yongshi and SARS-CoV-2 interactions

Initial complex models of HR1/Yongshi (2HR1/1Yongshi and 3HR1/3Yongshi) were built using SAdLSA sequence alignment and a crystal structure of HR1/HR2 complex (PDB 6LXT) as the template. Full atoms were modeled by Scwrl4^60^ and VMD^61^. Each structure was explicitly solvated in a water box, neutralized with additional ions, and minimized for 2000 conjugate gradient steps with NAMD version 2.14^62^ and CHARMM36 forcefields^63^. The 3HR1/3Yongshi complex was further equilibrated by 100 ps equilibration under constant pressure and temperature conditions (NPT) and followed by 100ns equilibration under constant volume and temperature conditions (NVT). An integration time step of 2fs was employed. Full electrostatics were computed using the particle-mesh Ewald (PME) method^64^. The calculations were done on a workstation with 24 Intel Xeon Gold 6226 CPU cores and 4 Nvidia RTX6000. Run time is 62 hours for the MD simulation.

### Biolayer interferometry

Biotinylated HR1 peptides from SARS-CoV-1, SARS-CoV-2, and MERS-CoV were synthesized by Fmoc synthesis and reconstituted in 100% DMSO. SARS-CoV-2 HR2, Yongshi, and D-Yongshi were reconstituted to 1 mM in DMSO. Bio-layer interferometry (BLI) experiments were performed on an Octet QK^e^ instrument at 25 °C with plate mixing at 1000 rpm. Peptide solutions were prepared in black non-binding plates (Greiner Bio-One, Monroe, NC) and binding responses to coated biosensors were recorded in manufacturer-supplied Data Acquisition software v11.1.1.19 (Sartorius).

All proteins were diluted in a running buffer of 20 mM HEPES, 150 mM NaCl, 0.02% Tween-20, pH 7.4. Biotinylated HR1 peptides were loaded to streptavidin sensors at 5 μg/mL to a threshold of 1.5 nm. After washing in the running buffer, the loaded sensors were moved to serial dilutions of SARS-CoV-2 HR2, Yongshi, or D-Yongshi for 100 s of association and 200 s of dissociation in running buffer. Sensors were depleted of remaining bound molecules by 3 rounds of 5 s washings in 500 mM NaCl followed by 5 s washings in running buffer.

A heterogenous ligand binding model was fit to the collected data in Data Analysis HT software v11.1.1.39 (Sartorius). Raw data and fittings were exported to GraphPad Prism v9.1. Approximate steady state calculations were performed by plotting response at the end of association to the concentration of protein used and fit to a hyperbola.

### Supplementary Information

Supplementary Table 1.

Supplementary Figures.

## Data Availability

All data generated or analyzed in this study are contained in the published article and supplementary materials.
