# Supplementary material for: A wild boar cathelicidin peptide derivative inhibits severe acute respiratory syndrome coronavirus-2 and its drifted variants
Source: Sci Rep. 2023 Sep 5;13:14650. doi: 10.1038/s41598-023-41850-7 (PMC10480232; doi:10.1038/s41598-023-41850-7)
Supplement: Supplementary file 1 — Supplementary Table 1. [file 41598_2023_41850_MOESM1_ESM.docx]

**Supplemental Information**

| Peptide Name | Sequence | Species | NCBI/Uniprot Reference Sequence |
| --- | --- | --- | --- |
| **PMAP-36R** | VGRFRRLRKKTRKRLKKIGKVLKWIPPIVGSIPLGCG | *Sus Scrofa* | NP_001123437.1 |
| **p9N** | KTRKRLKKIGKVLKWIPPIVGSIPLGCG | Truncation of PMAP-36R |  |
| p12N | KRLKKIGKVLKWIPPIVGSIPLGCG | Truncation of PMAP-36R |  |
| p14N | LKKIGKVLKWIPPIVGSIPLGCG | Truncation of PMAP-36R |  |
| **p2C** | VGRFRRLRKKTRKRLKKIGKVLKWIPPIVGSIPLG | Truncation of PMAP-36R |  |
| p5C | VGRFRRLRKKTRKRLKKIGKVLKWIPPIVGSI | Truncation of PMAP-36R |  |
| p9C | VGRFRRLRKKTRKRLKKIGKVLKWIPPI | Truncation of PMAP-36R |  |
| **Yongshi (pSer)** | VGRFRRLRKKTRKRLKKIGKVLKWIPPIVGSIPLGSG | Serine Mutant of PMAP-36R |  |
| **D-Yongshi** | (All D-amino acids) VGRFRRLRKKTRKRLKKIGKVLKWIPPIVGSIPLGSG | D-Enantiomer of Yongshi |  |
| **LL37** | LLGDFFRKSKEKIGKEFKRIVQRIKDFLRNLVPRTES | *Homo sapiens* | NP_004336.4 |
| OVA (257-264) | SIINFEKL |  | AUD54707.1 |
| SARS-CoV-2 HR2 | DISGINASVVNIQKEIDRLNEVAKNLNESLIDLQEL |  | P0DTC2 |
| SARS-CoV-1 HR1 | Biotin-ANQFNKAISQIQESLTTTSTALGKLQDVVNQNAQALNTLVKQ |  | P59594 |
| SARS-CoV-2 HR1 | Biotin-ANQFNSAIGKIQDSLSSTASALGKLQDVVNQNAQALNTLVKQ |  | P0DTC2 |
| MERS-CoV HR1 | Biotin-ANKFNQALGAMQTGFTTTNEAFQKVQDAVNNNAQALSKLASE |  | AXP07345.1 |
| PT | KRFKKFFMKLKKSVKKRVMKFFKKPMVIGVTFPF | *Pseudonaja textilis* | U5KJJ1.1 |
| PC1 | KRRKFFRSIRKRIKKLRKSIKKRLKKLPFEVPLVFSIPF | *Phascolarctos cinerus* | XP_020854988.1 |
| **PC 1a** | RKRIKKLRKSIKKRLKKLPFEVPLVFSIPF | Truncation of PC1 |  |
| CDC | KRFKKFFKKVKKSVKKRLKKIFKKPMVIGVSIPF | *Crotalus durissus cascavella* | AGS36137.1 |
| **NS** | KRFKNFFKKIKTGIKKVIKKTKE | *Notechis scutatus* | XP_026545399.1 |
| PB | KQPKRVKRFKKFFRKIKKGFRKIFKKTKIFIGGTIPI | *Python bivittatus* | XP_007443270.1 |
| **TS** | KRFKKFFKKIKKSVKKRVKKLFKKPRVIPISIPF | *Thamnophis sirtalis* | XP_013912467.1 |
| **Pangolin 1** | RTKRFRKLGNLLQKGGQKIGQKIERIGQKIKDFFSNLVPRQEGA | *Manis javanica* | XP_017524805.2 |
| Pangolin 1a | NLLQKGGQKIGQKIERIGQKIKDFFSNLVPRQEGA | Truncation of Pangolin 1 |  |
| Pangolin 1b | KIERIGQKIKDFFSNLVPRQEGA | Truncation of Pangolin 1 |  |
| Pangolin 1c | RTKRFRKLGNLLQK | Truncation of Pangolin 1 |  |
| Bat Cathelicidin 2 | VRRKETRAWRLPAPIGLWAPPGDGPAEPDPPVHGL | *Myotis lucifugus* | G1PZ56 |
| Bat Cathelicidin 2a | VRRKETRAWRLPAPIGLWAPPGDG | Truncation of Bat Cathelicidin 2 |  |
| Bat Cathelicidin 3 | VRFNYDRLSNIIKRGGYKLGEGLEIVG | *Myotis lucifugus* | XP_006108362.1 |
| Bat Cathelicidin 3a | VRFNYDRLSNIIKRGGYKLGEG | Truncation of Bat Cathelicidin 3 |  |
| Bat Cathelicidin 3b | VRFNYDRLSNIIKRGGYKL | Truncation of Bat Cathelicidin 3 |  |
| **Bat Cathelicidin 4** | IKNVELNIENLGERIKNAKKKVWEKIKSFGRRIKDFFRKPSPEVEP | *Myotis brandtii* | XP_014395994.1 |
| Bat Cathelicidin 4a | VELNIENLGERIKNAKKKVWEKIKSFGRRIKDFFRKPSPEVEP | Truncation of Bat Cathelicidin 4 |  |
| Bat Cathelicidin 4b | VELNIENLGERIKNAKKKVWEKIKSFGRRIKDFFRK | Truncation of Bat Cathelicidin 4 |  |
| **Bat Cathelicidin 4c** | GERIKNAKKKVWEKIKSFGRRIKDFFRKPSPEVEP | Internal Deletion of Bat Cathelicidin 4 |  |
| Bat Cathelicidin 4d | GERIKNAKKKVWEKIKSFGRRIKDFFRK | Truncation of Bat Cathelicidin 4 |  |
| Bat Cathelicidin 4e | GERIKNAKKKVWEKIKSFGRR | Truncation of Bat Cathelicidin 4 |  |
| Bat Cathelicidin 4f | KNVELNIENLGERIKNAKKK | Truncation of Bat Cathelicidin 4 |  |
| Bat Cathelicidin 5 | IDRSKLPPAVRDLYDNARNNIINNILRNF | *Myotis lucifugus* | XP_014306451.1 |
| Bat Cathelicidin 5a | AVRDLYDNARNNIINNILRNF | Truncation of Bat Cathelicidin 5 |  |
| Bat Cathelicidin 9 | GVPAQEPVRKAPPPPPDLQPQTPGPWPSPTWSPFPPPAQNKDFGFNSKR | *Myotis lucifugus* | G1Q810 |
| Bat Cathelicidin 9a | PVRKAPPPPPDLQPQTPGPWPSPTWSPFPPPAQNKDFGFNSKR | Truncation of Bat Cathelicidin 9 |  |
| Bat Cathelicidin 9b | PVRKAPPPPPDLQPQTPGPWPSPTWSPFPPP | Truncation of Bat Cathelicidin 9 |  |
| Bat Cathelicidin 11a | ERQQEDSREQPTAPPPEEEAASDIDR | *Myotis brandtii* | XP_005867266.1 |
| Bat Cathelicidin 13 | VILGAENLGERIKNAKKKVWEKIKSFGRRIKEFFRKPSPEVEP | *Myotis davidii* | ELK24988.1 |
| Bat Cathelicidin 13a | VILGAENLGERIKNAKKKVWEKIKSFGRRIK | Truncation of Bat Cathelicidin 13 |  |
| Cat 1 | RKLGQLGELIQQGGQKIVEKIQKIGQRIRDFFSNLRPRQEA | *Felis catus* | NP_001191707.1 |
| Cat 1a | KIVEKIQKIGQRIRDFFSNLRPRQEA | Truncation of Cat 1 |  |
| Cat 1b | KIGQRIRDFFSNLRPRQEA | Truncation of Cat 1 |  |
| **Beluga 1** | RRIPFWPIPLRWQWPPPWFPPSFPIPRISRKR | *Delphinapterus leucas* | XP_022428982.1 |
| Beluga 1a | PPPWFPPSFPIPRISRKR | Truncation of Beluga 1 |  |
| Puma 1 | LGQLGELIQRGGQKIGEKIQNIGQRIRDFFSNLRPMQEA | *Puma concolor* | XP_025772022.1 |
| Puma 1a | KIQNIGQRIRDFFSNLRPMQEA | Truncation of Puma 1 |  |

**Supplemental Table 1**. Sequence of peptides used in this study. Those with greater than 50% inhibition at 50µM appear in bold text.
