# Supplementary material for: A wild boar cathelicidin peptide derivative inhibits severe acute respiratory syndrome coronavirus-2 and its drifted variants
Source: Sci Rep. 2023 Sep 5;13:14650. doi: 10.1038/s41598-023-41850-7 (PMC10480232; doi:10.1038/s41598-023-41850-7)
Supplement: Supplementary file 2 — Supplementary Figures. [file 41598_2023_41850_MOESM2_ESM.pdf]

Supplemental Figure 1

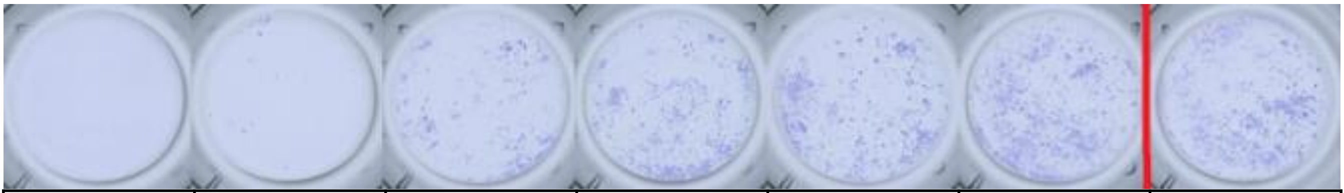

**25μM**	**12.5μM**	**6.25μM**	**3.12μM**	**1.56μM**	**0.78μM**	**Control**

Supplemental Figure 1. Representative IHC Staining of Vero E6 hACE2 cells infected with SARS-CoV-2 and treated with pSer. pSer at the labeled concentrations was pre-incubated with 100 pfu of live SARS-CoV-2 virus (nCoV/USA_WA1/2020) for 1 hour at 37° C before addition to confluent Vero E6 hACE2 cells in a 96-well plate. Infected cells were fixed, permeabilized, and stained with anti-spike antibody.

Supplemental Figure 2

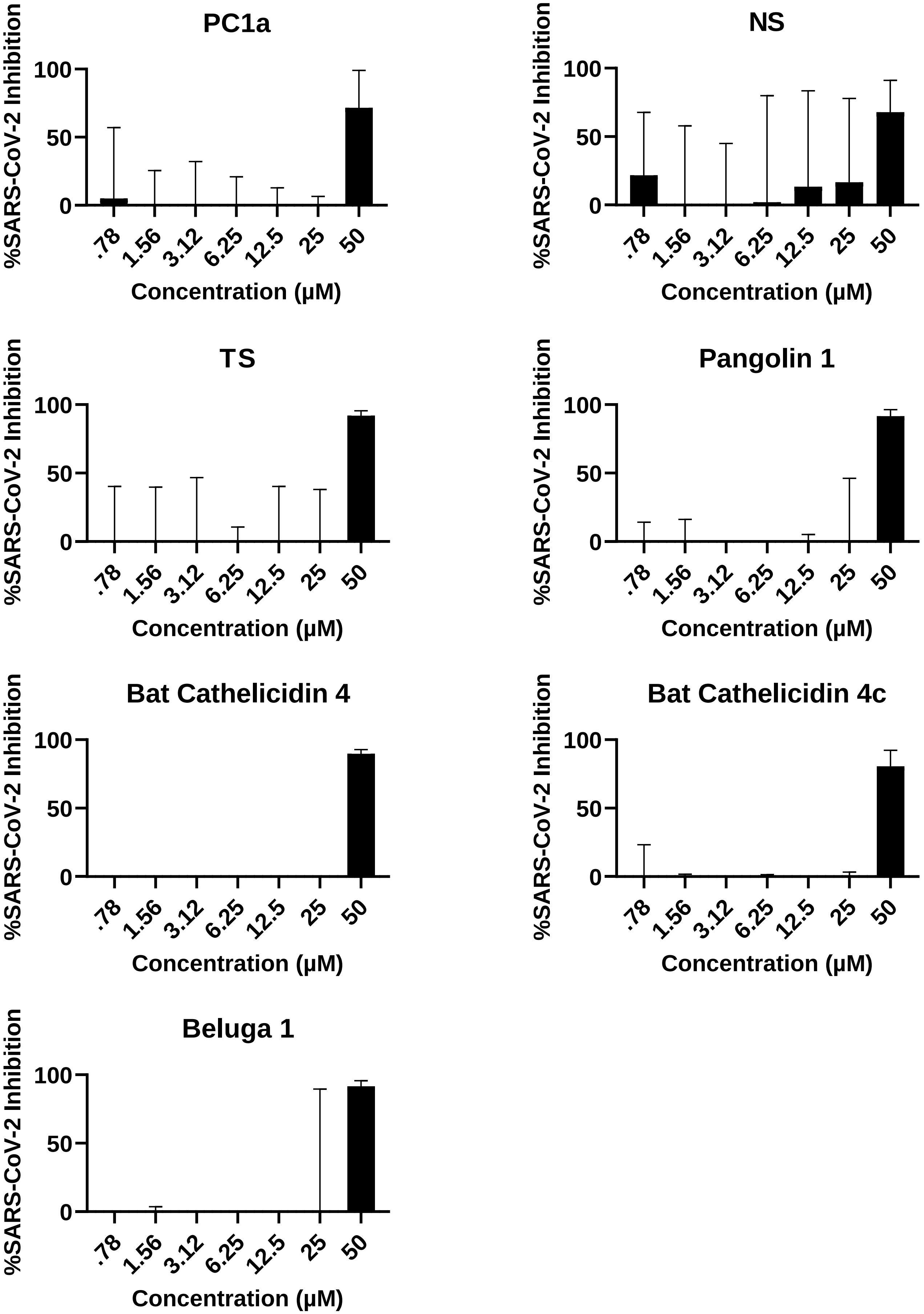

Supplemental Figure 2. Dose-response curves of other cathelicidin peptides with greater than 50% SARS-CoV-2 inhibition at 50µM. Inhibition of SARS-CoV-2 infection by various zoonotic cathelicidin peptides. Peptides at the labeled concentrations were pre-incubated with 100 pfu of live SARS-CoV-2 virus (nCoV/USA_WA1/2020) for 1 hour at 37° C before addition to confluent Vero E6 hACE2 cells in a 96-well plate. Infected cells were fixed and quantified by focus forming assay after 48 hours. Inhibition of viral infection was calculated based on the percent area of each well staining positively for viral spike protein compared to control wells without peptide inhibitor treatment. Results are representative of 3 independent experiments performed in triplicate.
